# Reward-Related Dysfunctions in Children Developing Attention Deficit Hyperactivity Disorder—Roles of Oppositional and Callous-Unemotional Symptoms

**DOI:** 10.3389/fpsyt.2021.738368

**Published:** 2021-10-22

**Authors:** Susan Schloß, Friederike Derz, Pia Schurek, Alisa Susann Cosan, Katja Becker, Ursula Pauli-Pott

**Affiliations:** ^1^Department of Child and Adolescent Psychiatry, Psychosomatics and Psychotherapy, Philipps-University of Marburg, Marburg, Germany; ^2^Center for Mind, Brain and Behavior, University of Marburg and Justus Liebig University Giessen, Marburg, Germany

**Keywords:** ADHD, externalizing disorders, callous-unemotional traits, developmental pathways, cognitive control, neurocognitive markers

## Abstract

**Objectives:** Neurocognitive functions might indicate specific pathways in developing attention deficit hyperactivity disorder (ADHD). We focus on reward-related dysfunctions and analyze whether reward-related inhibitory control (RRIC), approach motivation, and autonomic reactivity to reward-related stimuli are linked to developing ADHD, while accounting for comorbid symptoms of oppositional defiant disorder (ODD), and callous-unemotional (CU) traits.

**Methods:** A sample of 198 preschool children (115 boys; age: m = 58, s = 6 months) was re-assessed at age 8 years (m = 101.4, s = 3.6 months). ADHD diagnosis was made by clinical interviews. We measured ODD symptoms and CU traits using a multi-informant approach, RRIC (Snack-Delay task, Gift-Bag task) and approach tendency using neuropsychological tasks, and autonomic reactivity via indices of electrodermal activity (EDA).

**Results:** Low RRIC and low autonomic reactivity were uniquely associated with ADHD, while longitudinal and cross-sectional links between approach motivation and ADHD were completely explained by comorbid ODD and CU symptoms.

**Conclusion:** High approach motivation indicated developing ADHD with ODD and CU problems, while low RRIC and low reward-related autonomic reactivity were linked to developing pure ADHD. The results are in line with models on neurocognitive subtypes in externalizing disorders.

## Introduction

Attention deficit hyperactivity disorder (ADHD) and oppositional defiant and conduct disorder (ODD/CD) frequently occur together—about 50% of ADHD cases also develop ODD/CD (1). Longitudinal research has revealed a common developmental progression from preschool symptoms of ADHD to comorbid symptoms of ODD/CD in childhood and adolescence (2, 3). In recent years, research on externalizing disorders has pointed to a further distinguishable, early-developing psychopathological dimension, i.e., so-called callous-unemotional (CU) traits. CU traits, which comprise reduced guilt and remorse, callousness, low empathy, and deficient prosocial emotions, overlap with the dimensions of ADHD and ODD/CD symptoms (2, 4).

ADHD, ODD/CD and CU traits have been found to be associated with diverse neurocognitive dysfunctions (1). As these dysfunctions might represent markers of etiological subtypes or predictors of specific developmental pathways, the question of whether a dysfunction is uniquely related to ADHD or pertains to a specific combination with comorbid symptoms is an important issue of research (5). However, longitudinal research on this issue is scarce, especially between preschool and school age.

### Reward-Related Inhibitory Control and Approach Tendency

Dysfunctional processing of reward has been found to be prevalent in ADHD and in externalizing disorders. The “trait impulsivity” model, for example, postulates that early emerging impulsivity is a crucial vulnerability factor (externalizing liability) and indicative of a developmental pathway from ADHD to ODD and other externalizing disorders such as CD and substance use disorder (6). The impulsivity concept combines the two components of high subcortically mediated (bottom-up) approach motivation and low top-down inhibitory (cognitive) control (IC) mediated by the forebrain (7–9). In a further model, Blair et al. (2) proposed that deficient decision making, which comprises a high risk of impulsivity, represents the lowest common denominator for conduct problems. Similar to the trait-impulsivity model, the dysfunction is thought to involve subcortical bottom-up processes and prefrontal top-down control, and to be present in children showing multiple facets of externalizing problems, including children with ADHD, ODD/CD, and CU traits.

There is broad empirical evidence of low reward-related IC (RRIC) and other executive function deficits in children with ADHD as well as those with ODD/CD (10, 11). Low RRIC, dysfunctional reward-related decision making, and “delay aversion” have been assumed to characterize an ADHD subtype (12) with comorbid ODD/CD symptoms (13–15). CU traits, however, have sometimes been assumed to be associated with a rather good inhibitory control capacity and fewer cognitive deficits (4, 16, 17), making it possible that children with ADHD symptoms and comorbid CU traits show fewer RRIC deficits. Research on this issue is sparse. In particular, there are very few studies on the association between CU traits and IC in the context of ADHD development.

### Reward-Related Autonomic Reactivity

There is relatively broad evidence that children with ADHD show cortical arousal deficits. Cognitive deficits of the disorder have been assumed to be caused by difficulties in regulating arousal according to situational demands (1, 18, 19). Cortical and peripheral sympathetic arousal are linked via the locus coeruleus and the brain norepinephrine system. Measures of sympathetic electrodermal activity (EDA) have thus been taken as indicators of the arousal regulation dysfunction in ADHD (18). Bellato et al. (18) systematically reviewed the results of 55 studies on autonomic nervous system function in ADHD, and found that children and adolescents with ADHD showed hypoarousal (indicated, e.g., by low EDA). The authors concluded, however, that reactivity to rewarding, emotional stimuli, as well as the role of comorbidity, have not yet been sufficiently studied.

It has been shown that low resting-state sympathetic arousal in children with ADHD can be caused by comorbid ODD/CD problems, low anxiety, and psychopathic personality traits (20, 21). These characteristics overlap with CU traits (4, 22). Studies analyzing sympathetic arousal (using the cardiac pre-ejection period) during reward-related tasks found associations with comorbid ODD: Tenenbaum et al. (23) compared healthy children and children with ADHD, ADHD+ODD, and ADHD+CD regarding their sympathetic activity during a risky decision-making task and found the lowest activity in children with ADHD+ODD. In a sample of children with ADHD, Beauchaine et al. (24) found that sympathetic activity during a rewarded simple-matching task was associated with parent-reported conduct behavior problems. However, Conzelmann et al. (25) compared the electrodermal reactivity (EDR) to neutral, positive, and aversive stimuli between unmedicated boys with ADHD and healthy controls, and found lower EDR in the boys with ADHD in all three conditions, irrespective of comorbidity. As these studies did not assess CU traits, it is possible that comorbid CU traits and/or ODD symptoms explain low arousal in response to reward-related tasks.

Based on the research reviewed above, we examined the following hypotheses: (a) Low RRIC is associated with developing ADHD. This association overlaps with (i.e., can be explained by) comorbid ODD symptoms. We do not hypothesize an overlap with CU traits, as this top-down control component of impulsivity might not be impaired in children with CU traits. (b) High reward-related approach behavior is associated with developing ADHD. This association can be explained by ODD symptoms and CU traits, as this bottom-up component of impulsivity can be expected to be common to all three psychopathological domains. (c) Low autonomic reactivity to reward-related stimuli is linked to ADHD. This association overlaps with ODD symptoms and CU traits.

## Materials and Methods

### Participants

A sample of 198 preschool children (115 boys, 58%) was recruited from childcare facilities. The children were 4–5 years old (T1; m = 58, s = 6 months) at the first assessment wave and 8 years old (T2; m = 101.4, s = 3.65 months) at the second wave. Inclusion criteria were: IQ>80, lack of motor and sensory disabilities, lack of chronic physical and mental diseases, no indication of a trauma experienced by the child, and no continuous pharmacological treatment. To determine eligibility, a telephone interview and a screening questionnaire on the ADHD symptoms of the child [FBB-ADHS-V by (26), see below] were used. Children with high ADHD symptoms were oversampled. Of the 198 children, 179 participated in the 8-years assessment (retention rate of 89%). There were no differences between children who participated in the 8-years assessment and those who dropped out with respect to gender (Chi2 = 0.22; *t* = 0.00) and age of the child, ADHD symptoms, symptoms of anxiety/depression, and oppositional symptoms of the child (t-scores between −1.78 and 0.96).

At T1, all children were medication naïve. At 8 years (T2), three children were medicated with methylphenidate, and were therefore excluded from the analyses of the 8-years data. Table 1 contains descriptive data of the sample. Parents gave their written informed consent to participate in the study, and received an expense allowance of 50 Euros at T1 and 70 Euros at the T2 assessment. The study was approved by the Ethics Committee of the Medical Faculty, University of Marburg.

**Table 1:** Description of the sample.

**Gender**	*n* (%)
Male	115 (58.1)
Female	83 (41.9)
Education level of mother	*n* (%)
No compl./basic education	21 (10.6)
Work qualification	74 (37.4)
High school	36 (18.2)
College	67 (33.8)
Education level of father	*n* (%)
Basic education	36 (18.2)
Work qualification	47 (23.7)
High school	42 (21.2)
College	67 (33.8)
(No reply)	6 (3.0)
**T1 assessment**	
Questionnaire scores	m (s, range)
FBB-ADHS-V parent	1.04 (0.5, 0–2.5)
FBB-ADHS-V teacher	0.75 (0.6, 0–2.5)
FBB-SSV (ODD scale) parent	0.57 (0.5, 0–2.3)
**T2 assessment**	
ADHD diagnosis (CAPA interview)	*n* (%)
Yes	31 (17.4)
No	147 (82.1)
No CAPA interview	1 (0.6)
Questionnaire scores	m (s, range)
FBB-ADHS parent	0.81 (0.6, 0–2.6)
FBB-ADHS teacher	0.61 (0.6, 0–2.8)
SDQ (behavior problems scale) parent	2.00 (1.94, 0–10)
SDQ (behavior problems scale) teacher	3.17 (1.89, 0–8)
FBB-SSV (ODD scale) mother	0.72 (0.6, 0–2.9)
FBB-SSV (ODD scale) father	0.74 (0.6, 0–2.6)
SDQ/APSD (CU score) parent	4.0 (2.6, 0–11)
SDQ/APSD (CU score) teacher	4.5 (3.6, 0–14)
FBB-SSV (CU scale) mother	0.20 (0.3, 0–2.6)
FBB-SSV (CU scale) father	0.23 (0.3, 0–1.6)

*ADHD, attention deficit hyperactivity disorder; ODD, oppositional defiant disorder; CU, callous-unemotional; m, mean, s, standard deviation; APSD, Antisocial Process Screening Device; FBB-ADHS, questionnaire ADHD; FBB-SSV, questionnaire conduct disorders; SDQ, Strengths and Difficulties Questionnaire*.

### Variables

#### Assessments at T1

Reward-related inhibitory control. RRIC was measured using the Snack-Delay task by Kochanska (27). In this task, the child is instructed to wait for the ringing of a bell before he/she can retrieve a sweet that is covered by a transparent cup. After a practice trial, six trials followed, with delay intervals between 10 and 40 s. Waiting vs. approach behaviors are scored (27). The task is widely used for the assessment of RRIC in ADHD and has shown good psychometric properties (28). In the present study, tasks were carried out and scored by trained investigators. Interrater reliability was checked in 20% of cases and proved to be very good (ICC = 0.99).

Approach motivation. The Stranger-with-Toys (SWT) task (29) was used to capture behavioral approach motivation, i.e., the tendency to immediately approach a rewarding stimulus while disregarding possible risks associated with the unfamiliarity of the adult and the situation. In the past, similar tasks have been used to measure “exuberance” (30). In the SWT task, the child sits at a table with one rather boring toy. A stranger enters the room, bringing along a transparent bag of interesting toys, which she successively unpacks and plays with while not attending to the child. After 3 min, she invites the child to play with her together with the toys and continues to talk kindly to the child for a further 2 min. The latency (seconds) until the child's first spontaneous utterance directed to the stranger is scored. The measure has proven to be highly stable (0.74 across 2 years), and to show significant associations with parent ratings of the child's approach vs. withdrawal behavior, observed approach behavior in peer interactions (29), and ADHD symptoms of preschool children (31). Interrater reliability (checked in 20% of cases) was very good (ICC = 0.90).

ADHD and ODD symptoms of the child. The ADHD scale of the Parental Account of Childhood Symptoms (PACS) interview in the modified preschool version (Pre-PACS) (32) was conducted with the mother. The preschool version of the PACS interview has demonstrated good psychometric properties, and has proven to be suitable for the assessment of ADHD symptoms as a dimensional variable (33). Parents and teachers completed the preschool version of the ADHD rating scale (FBB-ADHS-V) by (26). This questionnaire is suitable for capturing ADHD symptoms according to the DSM-5 and ICD-10, and has shown high reliability and validity. In the present study, dimensional ADHD symptom scores were summed up (after z-transformation). Cronbach's Alpha of this summary score was 0.63.

Parents completed the ODD rating scale of the questionnaire (FBB-SSV), which has also shown good psychometric properties (26).

Anxiety and depressive symptoms of the child. The Anxious/Depressed scale of the German version of the Child Behavior Checklist (CBCL4-18) by Döpfner et al. (34) was employed for control purposes. The scale shows significant associations with anxiety and emotional disorders, indicating good validity (34).

#### Assessments at T2

Reward-related inhibitory control. At T2, we conducted the Gift-Bag task by Kochanska (27). In this task, the experimenter places a red paper bag containing a gift for the child on the table in front of the child. The experimenter then leaves the room for 5 min. The child is instructed not to look while awaiting the experimenter's return with the mother. Approach behavior was scored in accordance with Kochanska (27). Interrater reliability (20% of cases) proved to be very good (Kappa = 1.0).

Approach motivation. The interview on attractive toys (Int-AT) task adapted from Asendorpf (29) was conducted. As in the preschool task, approach behavior is provoked by a series of attractive toys and has to override a mild obstacle introduced by the unfamiliarity of the experimenter and the situation. The child is told that he/she will receive a gift for participating, but that prior to this, an interview on the attractiveness of a series of toys has to be conducted by a colleague. After 3 min of waiting (with a small book), an unfamiliar adult enters the room and places six different toys in front of the child and asks six questions, with a break of 10 s between the child's answer and the next question. The latency in seconds until the child's first spontaneous utterance toward the experimenter is scored.

Autonomic reactivity. Arousal level and reactivity of the sympathetic nervous system can be validly measured by indices of the EDA (35). We analyzed the electrodermal reactivity to the six questions of the Int-AT task. Baseline EDA (3 min) was recorded before the Int-AT task. The procedure was videotaped. Video and EDA recordings were synchronized. The measurement of EDA followed the guidelines by Boucsein et al. (35) using a BioPac MP150 system. EDA was measured as skin conductance level (in microsiemens) with two silver-silver chloride (Ag/AgCl) disposable electrodes attached to the middle phalanges of the middle and ring finger of the non-dominant hand. The mean skin conductance level (SCL) during baseline was calculated. To assess the child's sympathetic reactivity, the mean amplitude of the SCRs elicited by the six questions of the Int-AT task was determined.

ADHD diagnoses. At T2, the ADHD diagnostic module of the Child and Adolescent Psychiatric Interview (CAPA) by Angold et al. (36) was conducted with the mothers. The CAPA is a well-validated, widely established clinical interview. Diagnoses were made according to the DSM-5. Of the 179 children, *n* = 31 (15.7%) received an ADHD research diagnosis. Parents and teachers completed the ADHD questionnaire (FBB-ADHS of DISYPS-III) by Döpfner and Görtz-Dorten (37). In the present study, the parent (*r* = 0.63, *p* < 0.001) and the teacher (*r* = 0.54, *p* < 0.001) ADHD questionnaire scores were significantly associated with the ADHD diagnosis.

ODD symptoms. For the assessment of ODD symptoms, mothers and fathers completed the oppositional symptoms scale (of the FBB-SSV questionnaire; DISYPS-III) by Döpfner and Görtz-Dorten (37). Teachers and mothers, moreover, completed the conduct problems scale of the Strengths and Difficulties Questionnaire (SDQ) (38). We created a dimensional ODD symptom score by summing up the z-transformed scores of the mother (SDQ and FBB-SSV), father (FBB-SSV), and teacher (SDQ) (r's between 0.37 and 0.77, Cronbach's Alpha: 0.83).

CU traits. CU traits were assessed using the “prosocial behavior” scale of the SDQ and the “callous-unemotional” scale of the Antisocial Process Screening Device (APSD) (39). Mothers and teachers completed these questionnaires. The items of the two scales have proven to validly capture CU traits in 4–9-year-old children (40–42). Additionally, mothers and fathers completed the CU scale (of the FBB-SSV; DISYPS-III) by Döpfner and Görtz-Dorten (37). In the present study, the mother, father and teacher CU scores correlated significantly (r's between 0.23 and 0.44). We built a composite score by summing up the z-transformed scores (Cronbach's Alpha: 0.67).

Further control variables. Symptoms of anxiety disorders and depression were assessed by use of the screen interview of the DISYPS-III by Döpfner and Görtz-Dorten (37). The verbal IQ of the child was estimated by two subtests (Similarities and Vocabulary) of the Wechsler Intelligence Scale for Children [WISC-IV; (43)].

### Analytic Strategy

Correlation coefficients among the study variables were calculated for descriptive purposes. In those cases where gender of child was significantly associated with a neuropsychological/physiological predictor variable, we adjusted for gender in all respective analyses.

To test the hypotheses on the associations of (a) T1 and T2 RRIC, (b) T1 and T2 approach tendency and (c) T2 electrodermal reactivity with T2 ADHD diagnosis, we conducted logistic regression analyses. In analyses (a) and (b), we adjusted for T1 ADHD symptoms (model 2) to assess whether the predictor variables predict T2 ADHD over and above T1 ADHD symptoms. In all analyses (a, b, c), ODD and CU symptoms were covaried in model 3. For control purposes we additionally adjusted for anxiety/depressive symptoms and the approximated verbal IQ of the child in model 4.

In a next step, for the significant predictor-ADHD links, we tested whether ODD and/or CU symptoms significantly explain this link (i.e., the common variance between the predictor variable with the T2 ADHD diagnosis). For this purpose, we partitioned the total predictor-ADHD link (common variance) into the direct link (common variance between predictor and ADHD not explainable by the comorbid dimension) and the indirect link (i.e., common variance explainable by the comorbid dimension) using ordinary least squares (OLS) regression, and tested these links using the bootstrapping method recommended by Preacher and Hayes (44). The path-analytic procedure is suitable for analyzing the role of third variables (e.g., mediators, confounders, suppressor variables) in relationships between two variables (45). Calculations were conducted using the SPSS macro “Indirect” (44) and IBM SPSS Statistics software (IBM Corp.).

## Results

### Reward-Related Inhibitory Control

Consistent with our hypothesis, the T1 Snack-Delay task and the T2 Gift-Bag task were significantly associated with the T2 ADHD diagnosis (Table 3A, model 1). Associations remained statistically significant after adjusting for the T1 ADHD symptoms score (Table 3A, model 2). We further hypothesized that the link between RRIC and ADHD is shared with ODD symptoms. However, the T1 Snack-Delay task was only marginally significantly associated with the T2 ODD score, and the T2 Gift-Bag task was not correlated with the T2 ODD score (Table 2). Moreover, covariation of ODD and CU scores (Table 3A, model 3) did not change the significant associations between the RRIC tasks and T2 ADHD diagnosis. Hence, the results indicate a unique association between low RRIC and ADHD.

**Table 2:** Correlations among the study variables.

		**2**	**3**	**4**	**5**	**6**	**7**	**8^a^**	**9^a^**	**10^a^**	**11**	**12**
1	ADHD-s T1	0.41^***^	0.60^***^	0.32^***^	0.23^**^	−0.20^**^	−0.22^**^	−0.28^***^	−0.05	−0.16	−0.21^*^	−0.16^*^
2	ADHD-d T2	–	0.36^***^	0.42^***^	0.35^***^	−0.24^**^	−0.23^**^	−0.24^**^	−0.23^**^	−0.22^*^	−0.08	−0.19^*^
3	ODD-s T1		–	0.45^***^	0.29^***^	−0.08	−0.15	−0.17^*^	−0.17^*^	−0.14	−0.17	−0.19^*^
4	ODD-s T2			–	0.63^***^	−0.14	−0.17^*^	−0.15	−0.14	−0.12	−0.17	−0.16^*^
5	CU-t T2				–	0.03	−0.12	−0.24^***^	−0.16^*^	−0.17	−0.02	−0.35^***^
6	Snack-delay task T1					–	0.10	0.05	0.18^*^	0.18	0.03	−0.02
7	Gift-bag task T2						–	0.11	−0.01	0.06	−0.03	0.12
8	SWT task T1^a^							–	0.20^*^	−0.04	0.07	0.14
9	Int-AT task T2^a^								–	0.10	−0.10	0.07
10	SCR^a^									–	0.35^***^	0.18
11	SCL-BL										–	0.06
12	Gender of child											–

a *Spearman's Rho coefficients; Significance*

*** *p < 0.001*;

** *p < 0.01*;

* *p < 0.05*;

*SWT, stranger with toys; Int-AT, interview on attractive toys; SCR, mean skin conductance response; SCL-BL, skin conductance level at baseline; ADHD, symptoms of attention deficit hyperactivity disorder; ADHD-d, diagnosis of attention deficit hyperactivity disorder; ODD-s, symptoms of oppositional defiant disorder; CU-t, callous-unemotional traits*.

**Table 3:** Prediction of 8-years ADHD by neuropsychological/psychophysiological variables.

**Block**		**R^2^_Nagelkerke_ (change)**	**Chi^2^** **(df) (change)**	***P* (change)**
**A. PREDICTION BY RRIC**				
**Model 1**				
1	Snack-delay task T1	0.11	11.06 (1)	0.001
2	Gift-bag task T2	0.18	7.04 (1)	0.008
**Model 2**				
1	ADHD symp. T1	0.30	31.57 (1)	0.000
2	Snack-delay task T1	0.33	4.34 (1)	0.037
3	Gift-bag task T2	0.37	4.31 (1)	0.038
**Model 3**				
1	ODD symp. T1; ODD symp. T2; CU symp. T2	0.29	29.90 (3)	0.000
2	Snack-delay task T1	0.38	10.33 (1)	0.001
3	Gift-bag task T2	0.41	3.92 (1)	0.048
**Model 4**				
1	ODD symp. T1; Anxiety/depressive symp. T1; ODD symp. T2; CU symp. T2; Anxiety symp. T2; Depression symp. T2; Verbal IQ T2	0.31	31.92 (7)	0.000
2	Snack-delay task T1	0.40	10.02 (1)	0.002
3	Gift-bag task T2	0.42	3.82 (1)	0.051
**B. PREDICTION BY APPROACH MOTIVATION**				
**Model 1**				
1	SWT task T1	0.06	6.00 (1)	0.014
2	Int-AT task T2	0.12	5.30 (1)	0.021
**Model 2**				
1	ADHD symp. T1	0.27	27.20 (1)	0.000
2	SWT task T1	0.28	0.86 (1)	0.353
3	Int-AT task T2	0.34	6.89 (1)	0.009
**Model 3**				
1	ODD symp. T1; ODD symp. T2; CU symp. T2	0.30	29.86 (3)	0.000
2	SWT task T1	0.32	2.22 (1)	0.136
3	Int-AT task T2	0.36	4.23 (1)	0.04
**Model 4**				
1	ODD symp. T1; Anxiety/depressive symp. T1; ODD symp. T2; CU symp. T2; Anxiety symp. T2; Depression symp. T2; Verbal IQ T2	0.33	33.08 (7)	0.000
2	SWT task T1	0.35	2.42 (1)	0.120
3	Int-AT task T2	0.39	4.54 (1)	0.033
**C. PREDICTION BY SCR VARIABLE**				
**Model 1**				
1	SCR amp T2	0.09	6.13 (1)	0.013
**Model 3**				
1	ODD symp. T2; CU symp. T2	0.21	14.27 (2)	0.001
2	SCR amp T2	0.30	6.12 (1)	0.015
**Model 4**				
1	ODD symp. T2; CU symp. T2; Anxiety symp. T2; Depression symp. T2; Verbal IQ T2	0.25	17.18 (5)	0.004
2	SCR amp T2	0.31	4.15 (1)	0.042

*RRIC, reward related inhibitory control; SWT, stranger with toys; Int-AT, interview on attractive toys; SCR, mean skin conductance response; ADHD, attention deficit hyperactivity disorder; ODD, oppositional defiant disorder; CU, callous-unemotional*.

### Approach Motivation

The T1 SWT task and the T2 Int-AT task were significantly associated with the T2 ADHD diagnosis (Table 3B, model 1) indicating high approach motivation in children with ADHD. After adjusting for T1 ADHD symptoms, the prediction by the T1 SWT task was no longer significant (Table 3B, model 2). This finding indicates significant common variance between ADHD symptoms and high approach motivation already at T1. We further hypothesized that the link between approach motivation and ADHD can be explained by comorbid ODD and CU symptoms. Adjustment for the ODD and CU scores led to a reduction in the associations of T1 and T2 approach motivation task with T2 ADHD (Table 3B, model 3). Next, we analyzed whether T2 ODD and CU scores explain the link between T1 SWT task and T2 ADHD (Table 4). The indirect links via the ODD and via the CU score were significant. After accounting for the indirect link via the comorbidity scores, the SWT task-ADHD association was no longer significant. Hence, T1 approach motivation predicted ADHD with comorbid ODD and CU problems. Regarding the link of the T2 Int-AT task with T2 ADHD, indirect effects by CU and ODD scores were not statistically significant (Table 4).

**Table 4:** Path-analytic estimation of the direct and indirect links of approach motivation with ADHD, ODD, and CU symptoms.

**Direct effect**	**C ß**	**c' ß**	**Indirect effects by:**	**ß bootstrap CI_**95**_ lower upper bound**	***p* <**
T1 SWT task – T2 ADHD	−0.49^**^	−0.37	T2 CU symp.	−0.41 to −0.03	0.05
	−0.48^*^	−0.37	T2 ODD symp.	−0.39 to −0.01	0.05
T2 Int–AT task – T2 ADHD	−0.52^*^	−0.52^*^	T2 CU symp.	−0.33 to 0.02	ns
	−0.52^*^	−0.49^*^	T2 ODD symp.	−0.33 to 0.04	ns

*Significance*,

** *p < 0.01*,

* *p < 0.05*;

*ns, not statistically significant; ß, standardized path coefficient; SWT, stranger with toys; Int-AT, interview on attractive toys; ADHD, attention deficit hyperactivity disorder; ODD, oppositional defiant disorder; CU, callous-unemotional*.

### Reward-Related Autonomic Reactivity

As expected, children with ADHD showed lower mean SCRs to the questions on the attractiveness of the toys than did the other children (Table 2; Table 3C, model 1). Mean SCRs were not significantly correlated with ODD symptoms and CU traits (Table 2). Adjusting for ODD and CU scores did not change the significant association with the ADHD diagnosis (Table 3C, model 3). Hence, low sympathetic reactivity to the stimuli was uniquely associated with ADHD.

For the purpose of comparison, we additionally assessed baseline SCL. As shown in Table 2, the score was significantly associated with preschool ADHD symptoms. Associations with preschool and school-age ODD symptom scores just failed to reach statistical significance (*p*'s < 0.10). Children with high symptoms showed a lower baseline/resting arousal level.

## Discussion

In the present study, it was analyzed whether reward-related dysfunctions and sympathetic arousal are linked to ADHD development, and whether or not these links can be explained by ODD symptoms and CU traits. We found low RRIC, measured at preschool and school age, to be uniquely related to ADHD. Preschool RRIC significantly predicted ADHD development. The association between preschool approach motivation with school-age ADHD was significantly and completely explainable by comorbid ODD symptoms as well as by CU traits. Children with ADHD showed low autonomic responses to reward-related stimuli. This link was unique for ADHD, i.e., could not be explained by symptoms of ODD or CU traits. We discuss these findings in greater detail in the following.

We expected that RRIC deficits in ADHD can be explained by comorbid ODD symptoms. Contrary to this expectation, however, we found that the school-age ADHD diagnosis was significantly and uniquely associated with preschool- and school-age low RRIC. The associations could not be explained by comorbid ODD symptoms or CU traits. This result appears to correspond with a recent model on conduct problem development by Waller et al. (22). In this model, three pathways are distinguished, of which an ADHD pathway is characterized by low cognitive control. Hence, in this early stage of ADHD development, low cognitive control in the reward-related context might rather uniquely pertain to ADHD.

Corresponding to our expectations, high approach motivation at preschool and school age was associated with ADHD. Moreover, as expected, ODD symptoms and CU traits significantly and completely explained the link between preschool approach motivation and ADHD. Hence, high approach motivation at preschool age might indicate risk for the development of comorbid ADHD/ODD symptoms/CU traits. Several models have proposed that impulsivity forms the basis of externalizing disorders (7, 15). Blair et al. (2) assumed that deficient decision making in the context of reward and punishment (implying risk of impulsivity) is common to ADHD, externalizing disorders and CU traits. In the present study, we assessed the tendency to approach a gratification while overriding signals of threat (due to unfamiliarity). Thus, it seems probable that the SWT task captures the respective neurocognitive dysfunction at an early developmental stage. Based on these findings, it might be worthwhile to cross-validate and further refine the neuropsychological assessment of approach motivation as a risk predictor of the comorbid pathway.

In line with our hypothesis, children with an ADHD diagnosis showed comparably low mean SCRs to the reward-related stimuli at the 8-years assessment. The associations were unique for ADHD. ODD and CU symptoms were not associated with low SCRs. This finding corresponds to the results of Conzelmann et al. (25), who reported that children with ADHD showed low SCRs (regardless of the emotional valence of the stimuli), which were not explained by comorbidity. Hypoarousal, i.e., low, dysregulated cortical arousal, is an etiologically significant dysfunction in ADHD. Low arousal is thought to be implicated in cognitive and attentional deficits due to difficulties in the regulation of wakefulness and alertness states according to environmental demands (1, 18, 19). Our finding appears to be in line with this perspective.

Our study has several strengths, including the consideration of neuropsychological and psychophysiological characteristics as well as different psychopathological domains in a longitudinal design; the multi-informant approach (mother, father, and teacher reports) to the assessment of comorbid symptoms; the use of dimensional scores reflecting the expression of comorbid symptoms; and the analysis of a sample with increased ADHD symptoms, allowing for a sensitive and reliable description of ADHD development. A limitation might be seen in the lack of measurement of autonomic reactivity to neutral and negative stimuli. Such a measurement would have facilitated the comparison with previous research. Moreover, in future research, it would be interesting to assess further neuropsychological, biological and psychosocial characteristics and to analyze the role of ADHD symptom presentation (i.e., inattention or hyperactivity/impulsive symptoms) in order to increasingly refine the characterization of the developmental pathways. Due to possible influences on our findings the overlap between RRIC and the concept of delay aversion should be analyzed. As a further limitation of our study, it is not possible to draw any causal inferences from the findings. The reward-processing dysfunctions might constitute intermediate phenotypes involved in the development of the specific psychopathological problems, or may merely be correlates of these problems. In either case, however, an identification of neuropsychological/physiological predictors or indicators of emerging psychopathological pathways can be useful for risk identification and the application of tailored interventions.

Taken together, we assessed the development of ADHD while accounting for ODD symptoms and CU traits between preschool and school age. In line with current theorizing, we found that high approach motivation was linked to ADHD with comorbid ODD/CU symptoms. Low reward-related cognitive control (RRIC) and low autonomic reactivity to reward-related stimuli were specific for ADHD and might reflect the arousal regulation dysfunction of the disorder at an early stage of development. Given the scarcity of longitudinal data in this area, our results need to be cross-validated. The neurocognitive variables might constitute age-specific markers of clinically more homogenous pathways.

## Data Availability Statement

The datasets presented in this article are not readily available because they are part of a ongoing longitudinal study. Requests to access the datasets should be directed to ursula.pauli-pott@med.uni-marburg.de.

## Ethics Statement

The studies involving human participants were reviewed and approved by Ethics Committee of the Faculty of Medicine of the University of Marburg, Marburg, Germany. Written informed consent to participate in this study was provided by the participants' legal guardian/next of kin.

## Author Contributions

AC, KB, FD, PS, and SS organized the database. UP-P performed the statistical analysis. SS wrote the first draft of the manuscript. SS and UP-P wrote sections of the manuscript. All authors contributed to conception, design of the study, contributed to manuscript revision, read, and approved the submitted version.

## Funding

This work was supported by the German Research Foundation (Deutsche Forschungsgemeinschaft, DFG, grant number Be2573/3-1,2) and the University Hospital Marburg (grant number: 17/2018 MR).

## Conflict of Interest

The authors declare that the research was conducted in the absence of any commercial or financial relationships that could be construed as a potential conflict of interest.

## Publisher's Note

All claims expressed in this article are solely those of the authors and do not necessarily represent those of their affiliated organizations, or those of the publisher, the editors and the reviewers. Any product that may be evaluated in this article, or claim that may be made by its manufacturer, is not guaranteed or endorsed by the publisher.
